# Immunophenotypic aberrant hematopoietic stem cells in myelodysplastic syndromes: a biomarker for leukemic progression

**DOI:** 10.1038/s41375-023-01811-5

**Published:** 2023-02-15

**Authors:** Margot F. van Spronsen, Diana Hanekamp, Theresia M. Westers, Noortje van Gils, Eline Vermue, Arjo Rutten, Joop H. Jansen, Birgit I. Lissenberg-Witte, Linda Smit, Gerrit J. Schuurhuis, Arjan A. van de Loosdrecht

**Affiliations:** 1 Department of Hematology, Amsterdam UMC, Vrije Universiteit Amsterdam, Cancer Center Amsterdam, Amsterdam, The Netherlands; 2 Department of Hematology, Erasmus MC Cancer Institute, University Medical Center Rotterdam, Rotterdam, the Netherlands; 3 Department of Hematology, Radboud University Medical Center, Nijmegen, The Netherlands; 4 Department of Epidemiology and Data Science, Amsterdam UMC, Vrije Universiteit Amsterdam, Amsterdam, Netherlands

**Keywords:** Myelodysplastic syndrome, Haematopoietic stem cells, Cancer stem cells

## Abstract

Myelodysplastic syndromes (MDS) comprise hematological disorders that originate from the neoplastic transformation of hematopoietic stem cells (HSCs). However, discrimination between HSCs and their neoplastic counterparts in MDS-derived bone marrows (MDS-BMs) remains challenging. We hypothesized that in MDS patients immature CD34^+^CD38^−^ cells with aberrant expression of immunophenotypic markers reflect neoplastic stem cells and that their frequency predicts leukemic progression. We analyzed samples from 68 MDS patients and 53 controls and discriminated HSCs from immunophenotypic aberrant HSCs (IA-HSCs) expressing membrane aberrancies (CD7, CD11b, CD22, CD33, CD44, CD45RA, CD56, CD123, CD366 or CD371). One-third of the MDS-BMs (23/68) contained IA-HSCs. The presence of IA-HSCs correlated with perturbed hematopoiesis (disproportionally expanded CD34^+^ subsets beside cytopenias) and an increased hazard of leukemic progression (HR = 25, 95% CI: 2.9–218) that was independent of conventional risk factors. At 2 years follow-up, the sensitivity and specificity of presence of IA-HSCs for predicting leukemic progression was 83% (95% CI: 36–99%) and 71% (95% CI: 58–81%), respectively. In a selected cohort (*n* = 10), most MDS-BMs with IA-HSCs showed genomic complexity and high human blast counts following xenotransplantation into immunodeficient mice, contrasting MDS-BMs without IA-HSCs. This study demonstrates that the presence of IA-HSCs within MDS-BMs predicts leukemic progression, indicating the clinical potential of IA-HSCs as a prognostic biomarker.

## Introduction

Myelodysplastic syndromes (MDS) are hematological disorders affecting predominantly the elderly population with an incidence estimated at 17–21 cases per 100.00 individuals aged 70 years and over [1, 2]. These disorders are notorious for their biological and clinical heterogeneity. Therefore, MDS patient care relies on the accuracy of risk stratification. Until now risk stratification has been performed by models that incorporate peripheral blood (PB) values, bone marrow (BM) blast counts and cytogenetic abnormalities to stratify MDS patients in numerous categories that reflect a composite endpoint, i.e. leukemic progression and all-cause mortality [3, 4]. However, MDS patients succumb due to MDS-related and MDS-unrelated conditions and 25% transforms to acute myeloid leukemia (AML) [5]. Therefore, one might question the benefit of discriminating the many different categories in current models. While the progress in the understanding of MDS pathogenesis has shed new light on risk stratification by incorporating additional parameters as molecular data, there is no single marker that identifies every MDS patient predisposed to leukemia [6–9].

Based on cytogenetic, genetic and epigenetic alterations found in MDS-BM, MDS phenotypes are considered to arise from impaired maturation and differentiation of CD34^+^CD38^−^ hematopoietic stem cells (HSCs) [10–17]. However, characterization of conceptual cancer stem cells in MDS using flow cytometry and in vivo assays remains challenging. Research on AML enabled the definition of leukemic stem cells (LSCs) by the principle that healthy tissue-derived HSCs neither express lineage-infidelity antigens nor overexpress myeloid markers, whereas these can be expressed on malignant stem cells. Up to now, numerous leukemia-associated aberrantly expressed markers have been recognized, including CD2, CD7, CD11b, CD22, CD33, CD44, CD45RA, CD56, CD123, CD366 (TIM3) and CD371 (Clec12A) [18–20]. By demonstrating leukemic engraftment potential upon xenotransplantation, the leukemia-initiating nature of AML-derived CD34^+^CD38^−^ cells expressing these aberrancies has been confirmed [21, 22]. Moreover, it has been shown that the BM-derived LSC burden is associated with treatment outcome in AML patients [23].

Using the immunophenotypic features of LSCs in AML, several researchers were able to discriminate neoplastic- from benign stem cells in MDS by demonstrating that CD25, CD44, CD45RA, CD47, CD123, CD366, CD371 and IL1RAP are aberrantly expressed by MDS-derived CD34^+^CD38^−^ cells [24–32]. The markers IL1RAP and CD123 have been suggested as markers for targeted therapy because of altered cytogenetic and metabolic properties of IL1RAP- and CD123-positive stem cells, respectively [27, 33]. Moreover, several studies showed that the presence of stem cells aberrantly expressing certain antigens in MDS, including IL1RAP, CD25, CD366 and CD371, is associated with high-risk disease [25, 27, 29]. Nevertheless, none of the aforementioned studies had adequate power to identify a relationship between the presence of aberrant stem cells and leukemic transformation in MDS patients.

In this study, we immunophenotypically characterized stem cells in MDS by applying the LSC tube previously designed at our laboratory for flow cytometric detection of LSCs in AML [19]. By combining six markers into one fluorescence channel, this eight-color assay enables detection of thirteen cell surface antigens in a single-tube approach. The study of such a high number of markers in a single-tube approach is an advantage since HSCs are rare and antigens expressed by MDS-derived HSCs are heterogeneous [24–32, 34]. We demonstrated that one-third of the MDS patients contained immunophenotypic aberrant stem cells and that their presence is associated with leukemic progression and human engraftment potential in immune-deficient mice upon in vivo xenotransplantation.

## Methods

### Patient samples

Twenty-four cryopreserved BM samples from MDS patients and 128 fresh BM samples from cytopenic patients suspected of MDS were analyzed at the Amsterdam UMC, location Vrije Universiteit, between 2015 and 2017. Twenty-three/152 cases were excluded (Fig. S1). Following the World Health Organization (WHO) guidelines, MDS and chronic myelomonocytic leukemia (CMML) was confirmed in 76 and 5 patients, respectively [35]. Additionally, 40 pathological controls (PC) and 8 patients with cytopenia of undetermined causes (inconclusive) were included. Ten normal bone marrows (NBMs) and one PC were collected from 11 cardiothoracic surgery patients after obtaining written informed consent. The total study population comprised 140 individuals. This study was conducted following the Helsinki Declaration and approved by the Medical Ethics Committee of the Amsterdam UMC, location Vrije Universiteit (research ethics protocols: VUmc 2014-100, VUmc 2019-3448).

### Flow cytometry

Flow cytometry was performed using the LSC tube (Table S1A) [19]. Being absent on normal stem cells, CD7, CD11b, CD22, CD56, CD366 (TIM3) and CD371 (Clec12a) were combined into the PE channel (further referred to as “Combi”). CD33, CD44 and CD123 were studied in separate channels because of their moderate expression on normal stem cells that is usually lower than on their neoplastic counterparts. Although absent on normal stem cells, CD45RA and CD371 were studied separately in all and 62 samples, respectively. CD45RA was included since its value was revealed after validation of the Combi-channel. CD371 became more interesting after confirmation of its role as therapeutic target while our study was running [29, 31]. Experimental procedures were carried out following standardized protocols to reduce technical noise (SI: sample preparation, flow cytometry).

### Gating strategy

The CD34^+^ compartment was divided into CD38^−^, CD38^dim^ and CD38^+^ cells, the latter including common myeloid progenitors (CMPs), granulocyte-monocyte progenitors (GMPs) and megakaryocyte-erythroid progenitors (MEPs, Fig. S2). Following the strategy for LSC identification, immunophenotypic normal hematopoietic stem cells (HSCs) defined as CD34^+^CD38^−^ and CD33^−/+^/CD44^−/+^/CD45RA^−^/CD123^−/+^/Combi^−^ were distinguished from immunophenotypic aberrant hematopoietic stem cells (IA-HSCs) defined as CD34^+^CD38^−^ and CD33^++^ and/or CD44^+/++^ and/or CD45RA^+^ and/or CD123^++^ and/or Combi^+^ (SI: Gating strategy of IA-HSCs) [19, 20, 36]. The presence of IA-HSCs was never based on only CD33 or CD44 because gradual overexpression of these markers may be challenging to interpret, especially in case of CD33 polymorphism. To account for an underestimation of IA-HSCs in samples with few CD34^+^CD38^−^ cells, a limit of detection (LoD) and quantification (LoQ) was set at 10 and 30 CD34^+^CD38^−^ cells, respectively. The LoQ was used for multivariate analysis.

### Genomic analysis

Genomic DNA was isolated from cryopreserved-thawed BM-derived mononuclear cells (BM-MNCs) using a DNA kit (Qiagen, Hilden, Germany) following the manufacturer’s protocol. Amplicons covering relevant regions in 27 genes frequently affected in MDS were used (Table S2). For next-generation sequencing, the NextSeq500 instrument (Illumina, San Diego, CA, USA) was used following the manufacturer’s protocol (300 cycles High Output sequencing kit, v2), resulting in 2 × 150 base pairs paired-end reads. Candidate mutations with a variant allele frequency of at least 1% were selected.

### In vivo xenotransplantation

Nine weeks old female NOD-scid gamma (NSG) mice were irradiated one day before tail vein injection of BM-MNCs from 10 MDS patients. Xenotransplants contained as many as BM-MNCs as possible to optimize conditions for human engraftment (median, range: 4.5 ∙ 10^6^, 0.5–5.0 ∙ 10^6^ MNCs; SI: xenotransplantation). Mice (5 per sample) were sacrificed at 20 weeks post-transplantation (human endpoint, *n* = 42) or earlier in case of signs of illness (*n* = 8). BM, PB and spleen were analyzed for engraftment using a modified LSC tube including CD3, CD19 and murine CD45 (Table S1B). Experiments were performed at the Amsterdam Animal Research Center of the VU University following a protocol (850-HEMA17-01) approved by the Institutional Animal Care and Use Committee.

### Statistical analysis

Mann–Whitney *U*- and Chi-square tests were applied for testing numerical data following a non-normal distribution and categorical data in contingency tables, respectively. Univariate survival analysis was performed using the Kaplan–Meier method with the log-rank test. Multivariate survival analysis was carried out using Cox regression with forward selection (*P*-value for entry was 0.100). The leukemia-free survival (LFS), event-free survival (EFS) and overall survival (OS) were defined as the number of months from the date of sampling until the date of leukemic transformation, disease progression (≥ 5% blast increase) and all-cause mortality, respectively. Patients undergoing induction chemotherapy or stem cell transplantation were censored at the date of treatment start. Harrell’s C-index was used to evaluate the prognostic performance, assessing the concordance between predicted probabilities and observed outcomes. Confidence intervals (CI) with 95% coverage were used and two-sided *P*-values ≤ 0.05 were considered statistically significant. Analyses were conducted with the Statistical Package for the Social Sciences version 22 and R version 3.4.

## Results

### One-third of the MDS patients have IA-HSCs

**Fig. 1:**
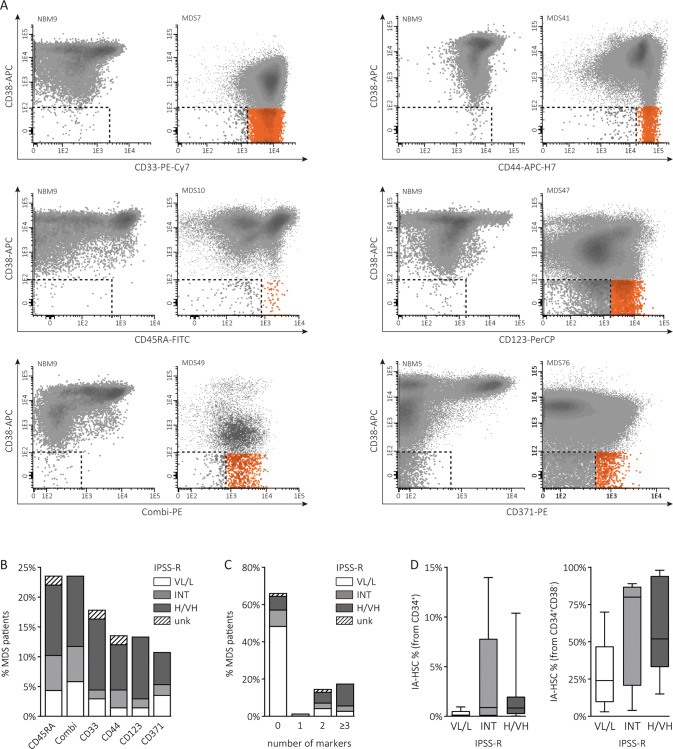
Characterization of IA-HSCs. **A** Flow cytometry plots demonstrating the total CD34^+^ HSPC compartment in a subset of NBM and IA-HSC^+^ MDS samples. To define HSCs (dotted outline) and IA-HSCs (orange), CD38^−^ cells were differentiated into CD33^-/+^/CD44^+^/CD45RA^−^/CD123^−^ versus CD33^++^ and/or CD44^++^ and/or CD45RA^+^ and/or CD123^++^ and/or Combi^+^ cells, respectively (Supplemental Information: Gating strategy of IA-HSCs). In samples with separate analysis of CD371, the Combi channel included CD7, CD11b, CD22, CD56 and CD366, but no CD371. In MDS7 and MDS41, almost all CD34^+^CD38^−^ cells overexpress CD33 and CD44, respectively. In MDS10, a clear CD45RA-negative population exists beside CD45RA-positive IA-HSCs. In MDS47, a large proportion of the CD34^+^CD38^−^ compartment overexpresses CD123. In MDS49, almost all CD34^+^CD38^−^ cells show positivity within the Combi channel. In MDS76, some CD34^+^CD38^−^ cells show aberrant expression of CD371. **B** The prevalence of IA-HSC markers presented as the percentage of MDS patients with CD34^+^CD38^−^ cells expressing that marker. For IA-HSCs^+^ MDS patients with more than one aberrancy, the figure displays all IA-HSC markers individually. The combination of IA-HSC markers is presented in Table 2. **C** The number of IA-HSC markers in MDS patients. Since we never defined IA-HSCs based on single overexpression of CD33, CD44 and CD123, IA-HSCs^+^ MDS-derived CD34^+^CD38^−^ cells often showed multiple aberrancies. **D** IA-HSC percentages defined by the best performing marker and relative from both CD34^+^ cells and CD34^+^CD38^−^ cells in IA-HSC^+^ MDS patients stratified by the IPSS-R. The boxplots indicate the median, the 25th and 75th percentile and upper and lower limits. VL/L very low- to low-risk, INT intermediate-risk, H/VH high- to very-high risk.

Our study comprised 76 MDS patients with a median age of 69.5 years (range, 37–89), a male predominance (75%) and distinct categories within the WHO classification, revised International Prognostic Scoring System (IPSS-R) and Comprehensive Cytogenetic Scoring System (CCSS) (Table 1). Using the LSC detection method validated in AML, we characterized aberrant marker expressions on CD34^+^CD38^−^ cells and defined aberrant maker-positive CD34^+^CD38^−^ cells as IA-HSCs (SI: Gating strategy of IA-HSCs) [19, 36]. We used a cluster of 10 CD34^+^CD38^−^ cells as threshold for immunophenotypic stem cell analysis, observed ≥10 CD34^+^CD38^−^ cells in 121/140 samples (Figs. S1–2) and defined samples with ≥10 IA-HSCs and <10 IA-HSCs as IA-HSC^+^ and IA-HSC^−^ samples, respectively. All NBMs (*n* = 9) and most PCs (*n* = 31/32) lacked aberrant antigen expressions on CD34^+^CD38^−^ cells. Contrarily, 23/68 (34%) MDS, 1/5 CMML and 3/7 inconclusive patients contained IA-HSCs (Fig. 1A). Data reanalysis and blinded analysis by a second researcher showed similar conclusions on the presence of IA-HSCs in 95% and 94% of the samples, respectively (*P* ≤ 0.001, Fig. S3). The CD34^+^CD38^−^ compartment from IA-HSCs^+^ MDS patients showed distinct immunophenotypes (Fig. 1B) and often more than one aberrancy (Fig. 1C). Most frequently expressed markers were CD45RA and markers within the Combi channel. Separate analysis of CD371 revealed that this antigen was the least often presented on CD34^+^CD38^−^ cells. We observed no relation between the risk stratification of MDS patients and the IA-HSC immunophenotype (data not shown). Based on its distinction between HSCs and IA-HSCs and its prevalence on CD34^+^CD38^−^ cells, we selected the best marker for each sample individually and used this marker to define IA-HSC percentages (Fig. 1D). Since the percentage of IA-HSCs varies per marker (Table 2), we further used the presence rather than the percentage of IA-HSCs in next analyses.

**Table 1:** MDS patient characteristics.

	All MDS *n* = 76	IA-HSC^-^ MDS *n* = 45	IA-HSC^+^ MDS *n* = 23	*P* value ^1^
Cell counts, median (range)				
Hemoglobin (g/dL)	9.7 (6.0–15)	10 (6–15)	8.8 (6.6–15)	0.020
Platelets (·10^9^/L)	104 (10–754)	163 (10–561)	59 (10–220)	<0.001
Neutrophils (·10^9^/L)	2.3 (0–10)	2.5 (0–10)	1.7 (0–8.7)	ns
White blood cells (·10^9^/L)	4.7 (0.5–35)	5.0 (1.5–35)	4.4 (0.5–22)	ns
PB blasts (%)	0 (0–9)	0 (0–9)	0 (0–3)	ns
BM blasts (%)	2 (0–17)	2 (0–17)	3 (0–16)	ns
WHO 2016, *n* (%)				ns
SLD	5 (6.6)	3 (6.7)	1 (4.3)	
MLD	22 (28.9)	14 (31.1)	5 (21.7)	
RS-SLD	2 (2.6)	2 (4.4)	0	
RS-MLD	25 (32.9)	14 (31.1)	9 (39.1)	
idel(5q)	1 (1.3)	0	1 (4.3)	
MDS-U	2 (2.6)	1 (2.2)	0	
EB-1	10 (13.2)	4 (8.9)	5 (21.7)	
EB-2	9 (11.8)	7 (15.6)	2 (8.7)	
IPSS-R, *n* (%)				<0.001
VL	10 (13.2)	8 (17.8)	0	
L	32 (42.1)	25 (55.6)	5 (21.7)	
INT	15 (19.7)	6 (13.3)	5 (21.7)	
H	9 (11.8)	5 (11.1)	4 (17.4)	
VH	8 (10.5)	0	8 (34.8)	
Missing	2 (2.6)	1 (2.2)	1 (4.3)	
CCSS, *n* (%)				<0.001
VL	1 (1.3)	1 (2.2)	0	
L	49 (64.5)	34 (75.6)	8 (34.8)	
INT	12 (15.8)	8 (17.8)	3 (13.0)	
H	3 (3.9)	1 (2.2)	2 (8.7)	
VH	10 (13.2)	0	10 (43.5)	
Missing	1 (1.3)	1 (2.2)	0	
Treatment, *n* (%)				<0.001
SC	42 (55.3)	31 (68.9)	6 (26.1)	
LEN	8 (10.5)	6 (13.3)	2 (8.7)	
AZA	8 (10.5)	2 (4.4)	6 (26.1)	
CTx	6 (7.9)	1 (2.2)	5 (21.7)	
SCT	8 (10.5)	3 (6.7)	4 (17.4)	
Missing	4 (5.3)	2 (4.4)	0	

The clinical characteristics are summarized for the entire MDS patient cohort and for MDS patients with >10 CD34^+^CD38^-^ cells categorized as IA-HSC^-^ or IA-HSCs^+^. ^1^ The *P-* value reflects the statistical significance between the clinical characteristics of IA-HSC^-^ versus IA-HSCs^+^ MDS patients. In one patient (MDS40), the percentage of blasts was based on a diagnostic flow cytometry panel because of poor quality of the BM smear.

*SLD* single lineage dysplasia, *MLD* multilineage dysplasia, *RS-SLD* single lineage dysplasia with ring sideroblasts, *RS-MLD* multilineage dysplasia with ring sideroblasts; isolated del(5q), MDS with isolated del(5q), *MDS-U* MDS unclassifiable, *EB-1* excess blasts type 1, *EB-2* excess blasts type 2, *SC* supportive care (including growth factors), *LEN* lenalidomide, *AZA* azacitidine, *CTx* chemotherapy, *SCT* stem cell transplantation.

**Table 2:** Summary of IA-HSC^+^ MDS patients.

	CD33	CD44	CD45RA	CD123	Combi	CD371	WHO	IPSS-R	CCSS	Tx	Event
	*n* = 11	*n* = 9	*n* = 16	*n* = 9	*n* = 16	*n* = 6					(month)
MDS28	6%	0%	22%	25%	24%	unk	MLD	L	L	CTx	AMLt (7)
MDS29	0%	0%	0%	0%	3%	1%	RS-MLD	L	INT	CTx	AMLt (6)
MDS39	24%	49%	0%	0%	0%	6%	del(5q)	L	L	LEN	SD (14)
MDS68	0%	0%	16%	0%	6%	unk	MLD	L	L	SC	PD (36)
MDS73	0%	0%	70%	0%	8%	unk	SLD	L	L	SCT	PD (3)
MDS9	70%	69%	51%	59%	85%	unk	EB-1	INT	L	AZA	PD (18)
MDS10	0%	80%	42%	0%	89%	unk	MLD	INT	VH	AZA	PD (6)
MDS17	0%	0%	0%	0%	37%	0%	RS-MLD	INT	L	SC	death (9)
MDS44	0%	unk	4%	0%	0%	3%	RS-MLD	INT	INT	GF	SD (1)
MDS49	0%	0%	37%	0%	80%	unk	EB-1	INT	L	GF	AMLt (20)
MDS18	unk	21%	15%	unk	36%	unk	MLD	H	H	SCT	SD (1)
MDS41	46%	93%	85%	61%	92%	1%	RS-MLD	H	INT	AZA	SD (5)
MDS50	95%	0%	0%	37%	0%	unk	EB-1	H	VH	AZA	death (9)
MDS7	98%	99%	0%	98%	0%	0%	EB-2	VH	VH	CTx	death (2)
MDS8	0%	0%	31%	0%	18%	unk	RS-MLD	VH	VH	CTx	PD (3)
MDS24	16%	26%	28%	36%	29%	0%	EB-1	VH	VH	AZC	SD (15)
MDS27	0%	0%	53%	0%	45%	unk	RS-MLD	VH	VH	CTx	AMLt (6)
MDS37	40%	37%	39%	38%	20%	0%	RS-MLD	VH	VH	SC	death (1)
MDS47	45%	0%	0%	48%	0%	8%	EB-1	VH	H	SCT	death (1)
MDS54	82%	0%	21%	68%	0%	0%	MLD	VH	VH	AZA	death (4)
MDS76	92%	0%	0%	0%	92%	11%	RS-MLD	VH	VH	LEN	death (7)
MDS35	0%	16%	16%	0%	0%	0%	EB-2	unk	L	SCT	unk
MDS74	0%	0%	31%	0%	51%	unk	RS-MLD	H	VH	SC	AMLt (4)

The percentages of IA-HSCs are relative to total CD34^+^CD38^−^ cells and depend on the selected marker. In the column “Event”, the number in brackets is the time in months after diagnosis when the outcome was assessed.

*UNK* unknown, *L* low-risk, *INT* intermediate-risk, *H* high-risk, *VH* very high-risk, *MLD* multilineage dysplasia, *RS-MLD* multilineage dysplasia with ring sideroblasts, del(5q), MDS with isolated del(5q), *EB-1* excess blasts type 1, *EB-2* excess blasts type-2, *Tx* treatment, *SC* supportive care, *GF* growth factors, *LEN* lenalidomide, *AZA* azacitidine, *CTx* chemotherapy, *SCT* stem cell transplantation, *SD* stable disease, *PD* progressive disease, *AMLt* transformation towards AML.

### The presence of IA-HSCs is associated with a disproportionate expansion of stem and progenitor cells

**Fig. 2:**
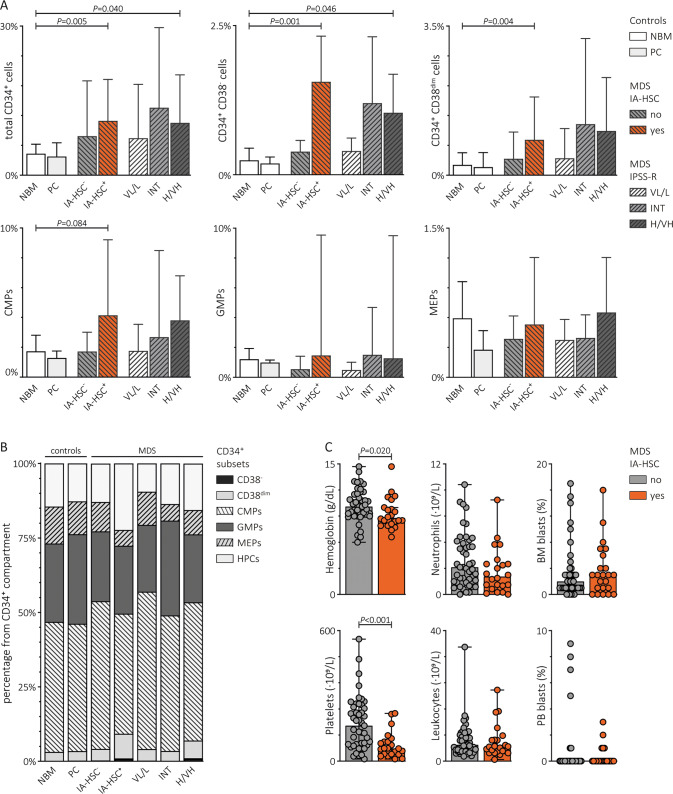
Bone marrow and peripheral blood counts in IA-HSC^+^ versus IA-HSC^−^ MDS patients. The Mann–Whitney *U* test was applied to test differences in the distribution of CD34^+^ subset numbers, blasts percentages and PB counts for statistical significance in controls compared to MDS patients stratified by the presence of IA-HSCs (IA-HSC^−^, *n* = 45; IA-HSC^+^, *n* = 23; <10 CD34^+^CD38^−^ cells, *n* = 8) and IPSS-R (VL/L, *n* = 42; INT, *n* = 15; H/VH, *n* = 17; unknown, *n* = 2). **A** The frequency of the total CD34^+^ compartment and CD34^+^ subsets relative to MNCs. The boxplots indicate median values with the 95% confidence interval. **B** The frequency of CD34^+^ subsets relative to the total CD34^+^ compartment. **C** The frequency of PB counts and blast percentages in IA-HSC^+^ versus IA-HSC^+^ MDS patients. VL/L very low- to low-risk, INT intermediate-risk, H/VH high- to very-high risk, HPCs CD34^+^CD38^+^ hematopoietic progenitors other than CMPs, GMPs and MEPs.

Questioning the role of IA-HSCs in MDS hematopoiesis, we quantified CD34^+^ subsets relative to MNCs in MDS patients stratified by the presence of IA-HSCs. To relate our results to previous findings, we additionally stratified patients by the IPSS-R (Fig. 2A). Comparable to IPSS-R very low- to low-risk and intermediate-risk, IA-HSC^−^ patients had similar CD34^+^ counts as NBMs. Contrarily, both IA-HSC^+^ patients (*P* = 0.005) and IPSS-R high- to very high-risk patients (*P* = 0.040) showed expanded CD34^+^ cells. Compared with NBM, IA-HSC^+^ patients had 12.6-fold, 5.5-fold and 2.4-fold increased CD34^+^CD38^−^ cells (*P* = 0.001), CD34^+^CD38^dim^ cells (*P* = 0.004) and CMPs (*P* = 0.084), respectively. Even IA-HSC^+^ patients classified as very low- to low-risk showed increased CD34^+^CD38^−^ cells (*P* = 0.031) and CD34^+^CD38^dim^ cells (*P* = 0.024) in comparison with IA-HSC^−^ patients from the same risk categories. Similarly, high- to very high-risk IA-HSC^+^ patients had increased CD34^+^CD38^−^ cells (*P* = 0.048) and a trend towards significantly increased CD34^+^CD38^dim^ cells (*P* = 0.064) than high- to very high-risk IA-HSC^−^ patients. Importantly, IA-HSC^+^ patients contained higher absolute CD34^+^CD38^−^ cell numbers but lower WBCs than IA-HSC^−^ patients, suggesting either a lower test sensitivity or disease-related CD34^+^CD38^−^ cell expansion in IA-HSC^−^ and IA-HSC^+^, respectively (Fig. S4). Quantification of CD34^+^ subsets relative to the total CD34^+^ compartment revealed disproportionate expansion of CD34^+^CD38^−^ (*P* = 0.013) and CD34^+^CD38^dim^ cells (*P* = 0.047) at the expense of MEPs (*P* *≤* 0.001) in IA-HSC^+^ patients compared with NBM, whereas the proportion of CD34^+^ subsets was preserved in IA-HSC^−^ patients (Fig. 2B). Next, we questioned whether the perturbed CD34^+^ composition in IA-HSC^+^ MDS co-occurred with an abnormal number of blasts and mature blood cells (Fig. 2C). Compared with IA-HSC^−^ patients, IA-HSC^+^ patients had comparable blast percentages but lower hemoglobin levels (*P* = 0.020) and platelet counts (*P* *≤* 0.001). Moreover, IA-HSC^+^ patients presented more often with pancytopenia (hemoglobin <10 g/dL, platelets <100 ∙ 10^9^/L, neutrophils <1.8 ∙ 10^9^/L) than IA-HSC^−^ patients (27% versus 6.7%, *P* = 0.020). These data suggest that the presence of IA-HSCs relates to MDS cases with perturbed immature hematopoiesis and severe cytopenias.

### The presence of IA-HSCs is highly predictive for progression from MDS to leukemia

**Fig. 3:**
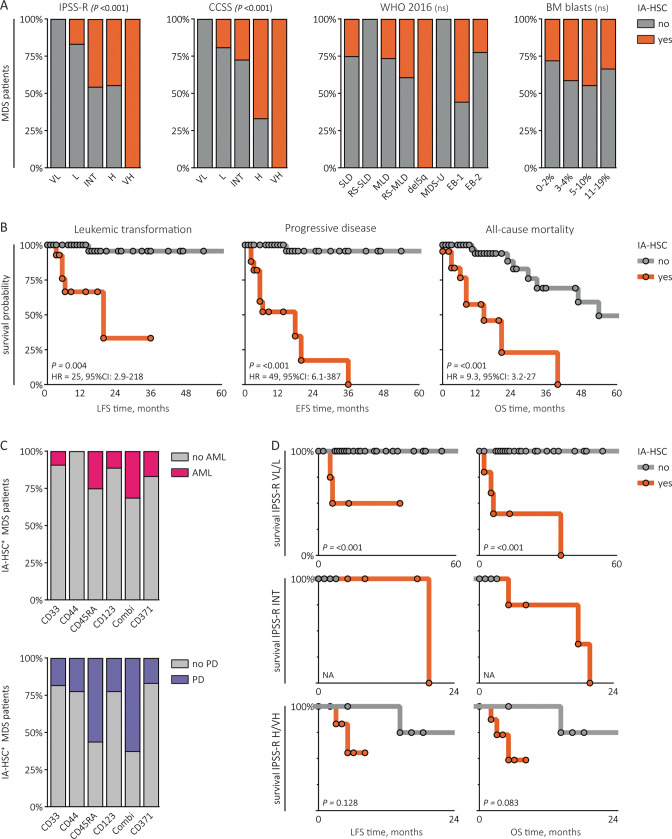
The prognostic value of the presence of IA-HSCs. **A** The distribution of IA-HSC^−^ and IA-HSC^+^ MDS patients over the IPSS-R, CCSS, WHO 2016 classification and BM blasts categories following IPSS-R cutoffs. **B** The LFS, EFS and OS times of IA-HSC^−^ and IA-HSC^+^ MDS patients. **C** The percentage of IA-HSC^+^ MDS patients with leukemic- and disease progression stratified by distinct IA-HSC markers. Among IA-HSC^+^ patients, patients with CD34^+^CD38^−^ cells expressing CD45RA or at least one Combi marker showed the highest prevalence of leukemic progression (25% and 31%) and disease progression (56% and 63%). **D** The LFS and EFS times of IA-HSC^−^ and IA-HSC^+^ MDS patients stratified by the IPSS-R. The log-rank test was not applied within intermediate risk IA-HSC^−^ and IA-HSC^+^ MDS patients considering the difference in FU time. VL/L very low- to low-risk, INT intermediate-risk, H/VH high- to very-high risk, PD progressive disease defined as a blast increase of ≥5%.

To explore the clinical value of IA-HSCs, we first studied the relation between the presence of IA-HSC^+^ and risk categories (Fig. 3A). The distribution of the WHO classification differed not significantly between IA-HSC^−^ and IA-HSC^+^ patients. According to the IPSS-R and CCSS, IA-HSC^+^ patients were at higher risk of a poor outcome than IA-HSC^−^ patients (both *P* *≤* 0.001). Nonetheless, following the IPSS-R and CCSS, 5/22 (23%) and 8/23 (35%) IA-HSC^+^ patients had a low-risk category whereas 5/22 (23%) and 3/23 (13%) IA-HSC^+^ patients had an intermediate-risk, respectively. Questioning the implications for survival, we studied the outcome of MDS patients stratified by the presence of IA-HSCs and IPSS-R. The presence of IA-HSCs correlated strongly to the clinical outcome (Fig. 3B, C). After a median follow-up time of 13 months (range 0.03-160), IA-HSC^+^ patients were at greater risk of leukemic transformation (HR = 25, *P* = 0.004), disease progression (HR = 49, *P* *≤* 0.001) and all-cause mortality (HR = 9.3, *P* *≤* 0.001) than IA-HSC^−^ patients. For predicting leukemic- and disease progression at two-year follow-up, the presence of IA-HSCs showed a sensitivity of 83% (95% CI: 36–99%) and 90% (95% CI: 54–99%) with a specificity of 71% (95% CI: 58–81%) and 76% (95% CI: 63–86%), respectively. Interestingly, one IA-HSC^+^ inconclusive patient and one IA-HSC^+^ CMML patient developed leukemia during follow-up (data not shown). We used univariate and multivariate Cox regression analyses to study the prognostic value of the percentage of IA-HSCs as a continuous variable and to assess the prognostic effect of the presence of IA-HSCs independent from treatment modalities and conventional risk factors, respectively (Table S3A, B). The presence of IA-HSCs was the most significant predictor of MDS patients’ outcome. The presence of IA-HSCs not only identified poor performing patients within IPSS-R high- to very high-risk, but also within the low-risk category (Fig. 3D). Moreover, the presence of IA-HSCs had a higher *C*-index for predicting leukemic (0.85 vs. 0.76) and disease progression (0.87 vs. 0.75) than the IPSS-R. These data suggest that the presence of IA-HSCs identifies MDS patients at risk of a progressive disease or leukemic evolution with additional value beyond the IPSS-R. Finally, we studied sequential BM samples in some MDS patients. Whereas patients with a stable disease (*n* = 2) maintained normal stem cell profiles, IA-HSC^+^ patients with a progressive disease (*n* = 4) showed IA-HSC expansion along with the increase in blasts (examples in Fig. S5). Moreover, low IA-HSC percentages persisted through induction chemotherapy or stem cell transplantation in relapsed patients (*n* = 3). These data suggest that IA-HSCs might have value to monitor disease progression and residual disease in MDS patients throughout the disease course similar to MRD assessments in AML.

### The presence of IA-HSCs may be associated with an adverse genomic background

Based on the unfavorable clinical outcome, we hypothesized that IA-HSC^+^ MDS have a more aggressive nature than IA-HSC^−^ MDS. To screen for mutational differences between IA-HSC^+^ and IA-HSC^−^ MDS, we performed sequencing on BM-MNCs in a subsampled cohort of 10 patients from distinct IPSS-R categories. We found no relationship between the presence of IA-HSCs and the number of mutations (Table S4). Yet, mutations of tumor suppressor genes and DNA methylation modification genes were unique for IA-HSC^+^ patients. Moreover, IA-HSC^+^ patients classified as IPSS-R low-risk (MDS73) and intermediate-risk (MDS9, MDS10, MDS17) displayed a relatively high genomic complexity corresponding with their unfavorable outcome (Table S2). Inversely, one IA-HSC^−^ patient (MDS43) had a favorable outcome despite the high-risk classification and numerous mutations. These observations suggest potential impact of IA-HSCs on survival besides the genomic background. Of course our sample size is too small to draw any conclusions.

### MDS-BMs with IA-HSCs give enhanced human engraftment in NSG mice

**Fig. 4:**
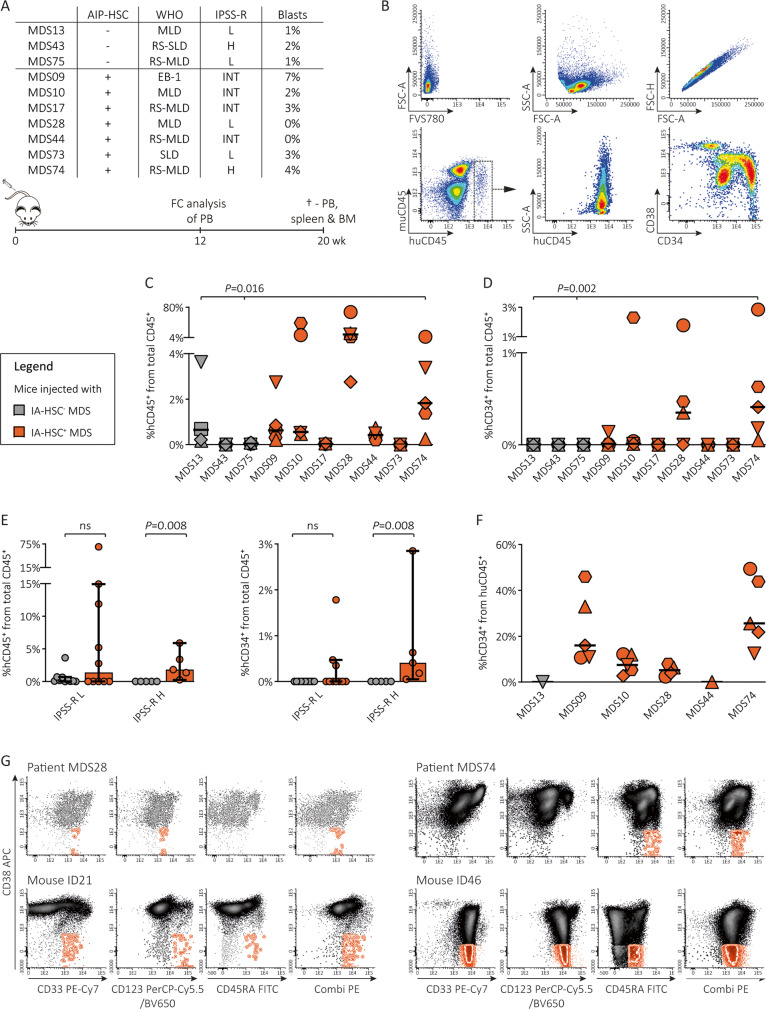
Xenotransplantation of BM-MNCs from IA-HSC^+^ versus IA-HSC^−^ patients. The percentages of huCD45^+^ and huCD34^+^ cells are enumerated from murine BM at 20 weeks post-transplantation and plotted as median with 95% CI. The Mann-Whitney U test was applied to test differences in the distribution of huCD45^+^ and huCD34^+^ cells between mice injected with IA-HSC^−^ and IA-HSC^+^ MDS samples as indicated by the grey and orange color, respectively. In the statistical analysis, the 5 mice per MDS patient were treated as unique measurements rather than averaged. **A** Summary of the experimental design: IA-HSC^−^ and IA-HSC^+^ MDS patients (*n* = 10) covering distinct IPSS-R risk groups were selected. Newborn NSG mice (*n* = 50) were sublethally irradiated and transplanted (*n* = 49) with BM from MDS patients. Engraftment of human cells was determined at 12 and 20 weeks post-transplantation within the PB and within the PB, spleen and BM, respectively. Despite the low blast count at diagnosis, patient MDS28 transformed to leukemia during disease progression and xenotransplantation of MDS28-derived BM-MNCs resulted into high huCD34^+^ engraftment numbers. **B** Flow cytometry plots illustrating the detection of huCD45^+^ cells and huCD34^+^ cells within murine BM (MDS74, mouse ID46). **C**, **D** The percentage of huCD45^+^ and of huCD34^+^ relative to the sum of mCD45^+^ and huCD45^+^ when excluding huCD3^+^ cells. Per MDS sample, the symbols represent the 4–5 individual mice injected with that sample. **E** The percentage of huCD45^+^ and huCD34^+^ within murine BM stratified by the IPSS-R risk groups of the MDS xenografts. IA-HSC^−^ low risk: MDS13, MDS75. IA-HSC^+^ low risk: MDS28, MDS73. IA-HSC^−^ high risk: MDS43. IA-HSC^+^ high risk: MDS74. **F** The percentage of huCD34^+^ cells within the BM from mice engrafting > 0.50% human cells. Per MDS sample, the symbols represent the 4–5 individual mice injected with that sample. **G** Flow cytometry plots illustrating the immunophenotype of huCD34^+^ cells within xenotransplanted mice in comparison with the original patient samples. Two examples are shown. Mouse ID21 had huCD34^+^CD38^−^Combi^+^CD33^+^CD123^+^ cells that also expressed CD45RA thereby contrasting with the original xenograft MDS28-derived IA-HSCs. Mouse ID46 propagated huCD34^+^CD38^−^ cells positive for CD45RA and the Combi channel similar to MDS10 patient-derived IA-HSCs. Moreover, CD33 and CD123 show a higher expression on murine-derived huCD34^+^ cells including huCD34^+^CD38^−^ cells than MDS10 patient-derived progenitors. BM bone marrow, PB peripheral blood, WHO World Health Organization, (RS-)MLD multilineage dysplasia (with ring sideroblasts), (RS-)SLD single lineage dysplasia, EB-2 excess of blasts type 2, IPSS-R revised International Prognostic Scoring System, L low risk, INT intermediate risk, H high risk.

To test whether the presence of IA-HSCs identifies MDS subtypes with leukemia-initiating potential, we transplanted BM-MNCs from IA-HSC^−^ (*n* = 3) and IA-HSC^+^ MDS patients (*n* = 7) into immunodeficient NSG mice (*n* = 5 for 9/10 samples and *n* = 4 for 1 sample, Fig. 4A and Fig. S6). Twenty weeks post-transplantation, we quantified human CD45^+^ (huCD45^+^) and CD45^+^CD34^+^ (huCD34^+^) cells using flow cytometry (Fig. 4B). Compared to IA-HSC^−^ MDS (1/3 engrafted), mice injected with IA-HSC^+^ MDS (4/7 engrafted) showed higher huCD45^+^ cell numbers within BM (*P* = 0.016, Fig. 4C), spleen (*P* = 0.003) and PB (*P* = 0.004, Fig. S7). Moreover, mice injected with IA-HSC^+^ MDS propagated higher huCD34^+^ cell numbers than IA-HSC^−^ transplanted mice (*P* *=* 0.002, Fig. 4D). The positive relationship between the presence of IA-HSCs and huCD45^+^ and huCD34^+^ engraftment in murine BM was maintained after stratification by the IPSS-R within the high-risk group (*P* = 0.008, Fig. 4E). Contrarily, we found no relationship between human engraftment numbers in murine BM and the IPSS-R or potential confounders including the number of injected BM-MNCs and remaining T cells as assessed by the Kruskal-Wallis test and linear regression analysis, respectively. Next, we enumerated huCD34^+^ cells relative to huCD45^+^ cells in murine BM with robust (≥ 0.50%) human engraftment. This revealed several IA-HSC^+^ transplanted mice with high huCD34^+^ cell numbers (Fig. 4F). Moreover, mice transplanted with 4/7 IA-HSC^+^ samples (MDS9, MDS10, MDS28, MDS74) contained huCD45^+^CD34^+^CD38^−^ cells with aberrant immunophenotypes (examples in Fig. 4G). Compared to patient-derived IA-HSCs, most mice harbored huCD45^+^CD34^+^CD38^−^ cells that expressed additional markers or lost markers, whereas few mice showed huCD45^+^CD34^+^CD38^−^ cells with a similar immunophenotype. Mice engrafted with MDS9 (CD33^+^CD123^+^CD45RA^+^Combi^+^ IA-HSCs) and MDS28 (CD45RA^+^CD123^+^Combi^+^ IA-HSCs) harbored huCD45^+^CD34^+^CD38^−^ cells with the similar immunophenotype (MDS9 *n* = 1, MDS28 *n* = 2) or with all markers except for CD45RA (MDS9 *n* = 3, MDS28 *n* = 1). Xenotransplantation of MDS10 (CD45RA^+^Combi^+^ IA-HSCs) resulted in outgrowth of huCD45^+^CD34^+^CD38^−^ cells positive for CD123 next to CD45RA and the Combi channel (*n* = 5). Mice transplanted with MDS74 (CD5RA^+^Combi^+^ IA-HSCs) demonstrated huCD45^+^CD34^+^CD38^−^ cells positive for the Combi channel next to CD33 and CD123 (*n* = 5) and loss of CD45RA (*n* = 2). Although leukemia characteristics may change upon outgrowth in vivo, these data suggest that IA-HSC^+^ MDS are distinct from IA-HSC^−^ in their potential to engraftment in immune-deficient mice, supporting the concept that the presence of IA-HSCs identifies MDS prone to leukemic evolution.

## Discussion

This study revealed that one-third of the MDS patients have aberrant expression of immunophenotypic markers on CD34^+^CD38^−^ cells referred to as IA-HSCs. The presence of IA-HSCs had prognostic value beyond current risk model, i.e. the IPSS-R, for predicting leukemic progression. Several IA-HSC^+^ patients classified as IPSS-R low-risk turned into MDS with excess blasts or AML, whereas some IA-HSC^−^ patients with IPSS-R high-risk had a stable disease course. Previous research on antigens differentiating between MDS-derived benign- and neoplastic stem cells failed to identify a relationship with leukemic progression presumably due to the focus on single markers [24–32]. Here, the use of the LSC tube enabled the simultaneous study of ten validated LSC markers on a higher number of CD34^+^CD38^−^ cells compared to what would have been possible in a multiple-tube approach [19]. This study suggests the feasibility of the LSC marker approach to identify MDS patients prone for a progressive disease or leukemic evolution with a high sensitivity (90% and 83%).

The cancer stem cell concept in MDS relies on several studies that revealed the existence of mutations within hematopoietic stem cells [14–17]. However, these studies defined the MDS stem cell compartment as Lin^−^CD34^+^CD38^−^CD45RA^−^ cells and thus depleted CD34^+^CD38^−^ cells aberrantly expressing CD45RA, CD7, CD11b, CD22 or CD56. We hypothesize that CD34^+^CD38^−^ cells with aberrant immunophenotypes play a critical role in leukemogenesis based on the relationship between the presence of IA-HSCs and leukemic transformation in MDS patients. Moreover, we showed that in vivo transplantation of BM-MNCs from IA-HSC^+^ MDS in contrast to IA-HSC^−^ patients resulted in human xenotransplants with high CD34^+^ counts and immunophenotypic aberrant CD34^+^CD38^−^ cells. In line with our data, Chen et al. showed that MDS-derived Lin^−^CD34^+^CD38^−^ cells aberrantly expressing CD45RA or CD123 are more biased towards myeloid differentiation upon xenotransplantation than their normal counterparts [37]. Jansen et al. recently performed genetic analysis of distinct immunophenotypically defined subclones and showed that some subclones with normal immunophenotypes harbor several genetic abnormalities, suggesting that genetic analysis is complementary to immunophenotyping in some cases [38]. Although the leukemic nature of immunophenotypically defined LSCs has been already demonstrated in AML, proof for the hypothesis that MDS-derived IA-HSCs are enriched for leukemic stem cells should rely on serial xenotransplantation studies and genetic analysis of immunophenotypically sorted HSCs and IA-HSCs [21, 22, 39].

Previous studies showed that low- and high-risk MDS are differentiated by increased CMPs and decreased GMPs versus increased stem cells and GMPs, respectively [14–17]. Differently, our results revealed normal frequencies in IA-HSC^−^ and IPSS-R very low- to low-risk patients whereas IA-HSC^+^ and IPSS-R high- to very high-risk patients showed disproportionate expansion of stem cell subsets, and to a lesser extent CMPs, at the expense of MEPs. The difference in progenitor numbers between our and previous studies could be explained by the method of cell calculation, gating strategy and study population. Nonetheless, our results confirm the finding of previous publications that disproportionate stem cell expansions, suggestive of uncontrolled self-renewal and impaired differentiation, distinguish indolent from advanced MDS subtypes as identified by IA-HSCs or the IPSS(-R).

Several issues are relevant to mention for the interpretation of our data. Our results are constrained by patients numbers, especially those with leukemic progression. Furthermore, the impact of IA-HSCs may have been relatively simple to detect since our study population over-presents high-risk MDS due to referral bias. A prospective multicenter population-based study is needed to confirm the predictive power of IA-HSCs in MDS. Recent work of Hanekamp et al. is a step ahead in the multi-center validation of manually gated LSCs in AML patients [36]. Whereas manual gating of low-frequent cell populations is sensitive to errors and a potential cause of intra- and inter-laboratory variability, this was rather restricted in our study. Future investigations might prove manual gating with computational identification of IA-HSCs as a robust assay that can be used universally [33, 34].

In conclusion, this study identified IA-HSCs as presumed neoplastic stem cells in one-third of the MDS patients using the knowledge of LSCs in AML. Results suggest the clinical utility of flow cytometric detection of IA-HSCs for early-stage prediction of MDS patients predisposed to leukemia. Considering the significance of this assay, we envision that the presence of IA-HSCs may be applied in the future to support risk assessment and guide treatment decisions in MDS patients. If IA-HSCs truly represents neoplastic stem cells, their aberrant antigen expressions may serve as targets for therapeutic intervention. To this end, an unbiased search for possible candidate markers of neoplastic stem cells in MDS is needed.

## Supplementary information

Supplemental Material

## Data Availability

The data that support the findings of this study are available on request from the corresponding author, A. A. van de Loosdrecht, upon reasonable request.
