# Supplementary material for: Immunophenotypic aberrant hematopoietic stem cells in myelodysplastic syndromes: a biomarker for leukemic progression
Source: Leukemia. 2023 Feb 15;37(3):680–90. doi: 10.1038/s41375-023-01811-5 (PMC9991914; doi:10.1038/s41375-023-01811-5)
Supplement: Supplementary file 1 — Supplemental Material [file 41375_2023_1811_MOESM1_ESM.pdf]

Immunophenotypic aberrant hematopoietic stem cells in myelodysplastic syndromes: a biomarker for leukemic progression

Table of Contents

Figure S1 Flowchart of the study population page 3

Sample preparation page 4

Flow cytometry page 4

Tables S1A-B LSC tubes page 5

Table S2 Sequencing panel page 6

Gating strategy of IA-HSCs page 7

Figure S2 Gating strategy page 8

Figure S3 Validation of the gating strategy page 9

Figure S4 Measured $CD34^+$ cell counts page 10

Table S3A-B Univariate and multivariate survival analyses page 11 - 12

Figure S5 Analysis of stem cells in sequential bone marrow samples page 13

Table S4 Mutations in BM-MNCs from a subsampled MDS patient cohort page 14

In vivo xenotransplantation page 15

Figure S6 Xenograft counts page 16

Figure S7 Human engraftment in spleen and peripheral blood page 17

Figure S1 Flowchart of the study population

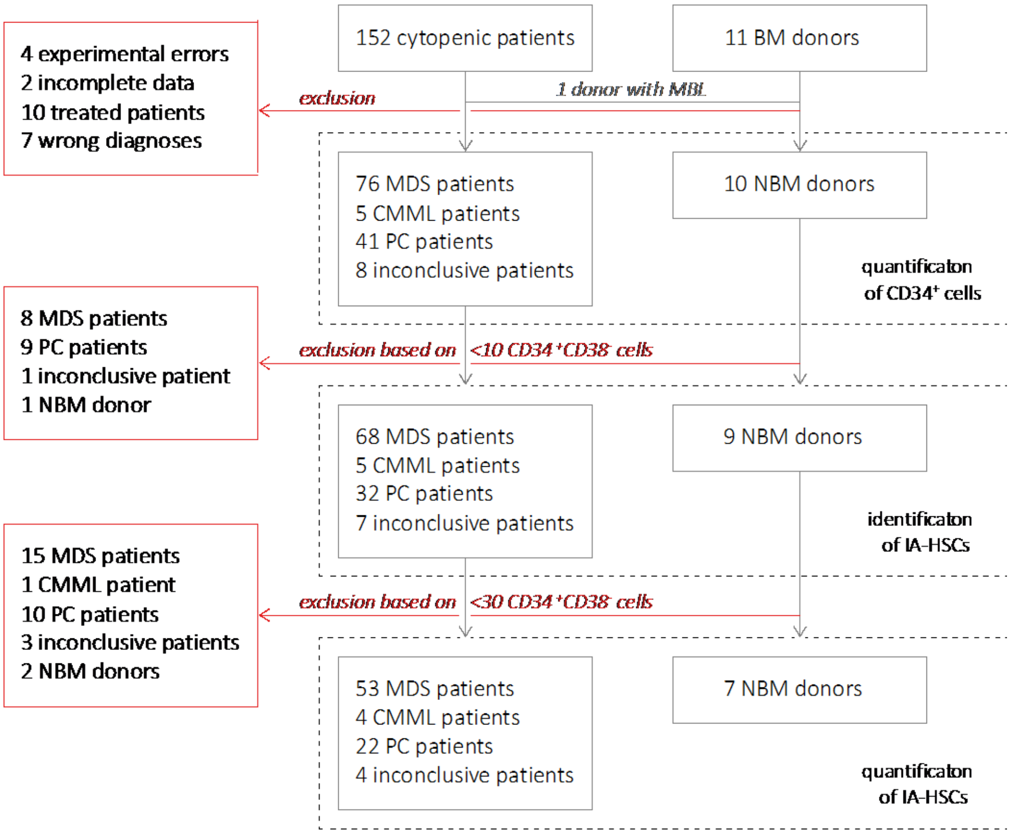

Normal bone marrow (NBM) was collected from age-matched cardiothoracic surgery patients (median age (range): NBM, 68 (62-80) years versus myelodysplastic syndromes (MDS), 70 (69-88) years). Patients with reactive conditions, nutritional deficiencies or non-myeloid clonal disorders were considered as pathological controls (PC). Twenty-three patients were excluded because of experimental errors (n=4), insufficient clinical data (n=2), a history of therapy for MDS before bone marrow (BM) sampling (n=10) or cytopenia due to non-MDS malignancy, including myelofibrosis (n=2), multiple myeloma (MM, n=1), acute myeloid leukemia (AML, n=1), acute erythroid leukemia (n=1), polycythemia vera (n=1) and Diamond-Blackfan anemia (n=1). Two patients with MDS in addition to MM were included and were previously treated for MM prior to bone marrow sampling. One BM donor was considered as PC because of the presence of monoclonal B cell lymphocytosis (MBL). Two patients with MDS in addition to MM were included. Quantitative analysis of the $CD34^+$ cell compartment was performed in all included patients. By defining the limit of detection (LoD) and limit of quantification (LoQ) at ≥10 and ≥30 $CD34^+CD38^-$ cells, the presence and the number of immunophenotypic aberrant hematopoietic stem cells (IA-HSCs) was determined in 121/140 and 90/140 samples, respectively.

Sample preparation

Bone marrow (BM) samples were analyzed fresh (82%) or after cryopreservation (18%). Fresh samples were deprived of erythrocytes using an ammonium chloride solution (Pharm Lyse, BD Biosciences) for 10 minutes at room temperature. Lysed samples were washed twice and resuspended in the washing solution (phosphate buffer saline (PBS) with 0.1% human serum albumin (HSA) and 0.05% sodium azide). Mononuclear cells (MNCs) were obtained using a Ficoll gradient (1.077 g/mL, Amersham Biosciences) and erythrocyte lysis using home-made ammonium chloride buffered lysing solution and then cryopreserved in RPMI-1640 (Gibco, Life technologies) with 20% heat-inactivated fetal bovine serum (FBS, Greiner) and 10% DMSO (Riedel-de Haën). Cryopreserved MNCs were thawed in a 37 °C water bath and recovered in preheated RPMI with 20% FBS, 0.1% DNAse (Sigma-Aldrich) and 0.1% $MgCl_2$ [1M]. Thawed MNCs were washed and resuspended in PBS with 0.1% HSA.

Flow cytometry

Prepared cells were incubated with the antibody mixture (Table S1A-B) for 30 minutes at room temperature and subsequently washed once with the washing solution. The marker CD371 was analyzed separate from the Combi channel in 62 samples, including NBM (n=1), MDS (n=35), chronic myelomonocytic leukemia (n=4), PC (n=19) and inconclusive cases (n=3). All antibodies were purchased from BD Biosciences, except for the CD366 antibody and the Fixable Viable Stain that were obtained from R&D Systems and Thermo Fisher Scientific Inc., respectively. Human and murine samples were measured on a BD FACS Canto II and a BD FACS Celesta, respectively. The quality control of the flow cytometers was performed daily and the compensation settings were periodically updated following a standardized protocol.

<u>Table S1A</u> The LSC tube

	Marker	Fluorchrome	Clone
1	CD45	HV500c	2D
2	CD34	BV421	581
3	CD38	APC	HB7
4	CD33	PE-Cy7	P67.6
5	CD44	APC-H7	G44-26
6	CD45RA	FITC	L48
7	CD123	PerCP-Cy5.5	7G3
	Combi		
8	CD7	PE	M-T701
9	CD11b	PE	D12
10	CD22	PE	S-HCL-1
11	CD56	PE	My31
12	CD366 (TIM3)	PE	344823
13	CD371 (Clec12a)	PE	50C1

The 8-color LSC tube includes common markers (CD45, CD34, CD38) next to lineage- and leukemia-associated markers. Antigens without expression on normal stem cells can be combined within the PE-channel (further referred to as “Combi”), since their cumulative expression on normal stem cells remains negative. CD45RA is absent on normal stem cells but studied separately as this marker was added later on, i.e. after having validated the Combi channel. CD33, CD44 and CD123 are expressed by normal stem cells and should therefore be studied separately to define overexpression. [1] All antibodies were purchased from BD Biosciences, except for the CD366 antibody and the Fixable Viable Stain that were obtained from R&D Systems and Thermo Fisher Scientific Inc., respectively.

<u>Table S1B</u> Adjustment of the LSC tube for engraftment analysis

Number	Marker	Fluorchrome	Clone
1	Fixable Viability Stain 780	APC-H7	
2	muCD45	PerCP	30-F11
3	huCD45	HV500c	2D
4	CD3	FITC	SK7
5	CD19	BUV737	SJ25C1
6	CD34	BV421	581
7	CD38	APC	HB7
8	CD33	PE-Cy7	P67.6
9	CD45RA	AF700	HI100
10	CD123	BV650	7G3
	Combi		
11	CD7	PE	M-T701
12	CD11b	PE	D12
13	CD22	PE	S-HCL-1
14	CD56	PE	My31
15	CD366 (TIM3)	PE	344823
16	CD371 (Clec12a)	PE	50C1

Table S2 Sequencing panel

Number	Gene	RefSeq	Exon
1	ASXL1	NM_015338	13 (partially)
2	BRAF	NM_004333.4	ENST00000288602
3	CALR	NM_004343	9
4	CBL	NM_005188	8-9
5	CSF3R	NM_156039	14, 17
6	DNMT3A	NM_175629	all coding exons
7	ETNK1	NM_018638	3 (partially)
8	EZH2	NM_004456	all coding exons
9	FLT3_835	NM_004119	20 (partially)
10	IDH1	NM_005896	4 (partially)
11	IDH2	NM_002168	4 (partially)
12	JAK2	NM_004972	14 (partially), 12
13	KIT	NM_000222	8 (partially), 17 (partially)
14	KRAS	NM_004985	2-3 (partially)
15	MPL	NM_005373	10 (partially)
16	MYD88	NM_002468.4	ENST00000417037
17	NOTCH1	NM_017617.4	ENST00000277541
18	NPM1	NM_002520	11 (partially)
19	NRAS	NM_002524	2-3 (partially)
20	RUNX1	NM_001754	all coding exons
21	SETBP1	NM_015559	4 (partially)
22	SF3B1	NM_012433	13-16
23	SRSF2	NM_003016	1 (partially)
24	TET2	NM_001127208	all coding exons
25	TP53	NM_000546	all coding exons
26	U2AF1	NM_006758	2, 6 (partially)

Gating strategy of IA-HSCs

Gating of leukemic stem cells (LSCs) in AML samples using the LSC tube has been extensively described by Hanekamp et al. [2] Similarly, the $CD34^+CD38^-$ stem cells and $CD34^+CD38^{dim}$ immature progenitor cells were selected as summarized in Figure S2. Lymphocytes were defined as $FSC^{low}/SSC^{low}/CD45^{++}$ (not shown) and used as an internal negative control (for CD33, CD44, CD123 and CD371) or positive control (for CD45RA and Combi) to determine the cut-off between normal stem cells and IA-HSCs. The $CD34^+CD38^{dim}$ cell compartment was additionally studied to substantiate our definition of IA-HSCs, considering that abnormal marker expressions on $CD34^+CD38^-$ cells may be passed onto downstream progenitors. All markers (i.e. CD33, CD44, CD123, CD45RA and Combi) were gated separately on $CD34^+CD38^-$ cells. Generally, IA-HSCs were covered by more than one marker as shown in Figure 1. Therefore, the best marker was selected to define the IA-HSCs load, presented as the percentage out of distinct reference categories, including $CD34^+CD38^-$ cells, total $CD34^+$ cells and MNCs. Since we have shown before that aberrant antigen expression on both $CD34^+CD38^+$ cells [3] and $CD34^+$ $CD38^-$ cells [4] are not affected by the freeze-thawing procedure, we used fresh as well as cryopreserved-thawed samples in our study.

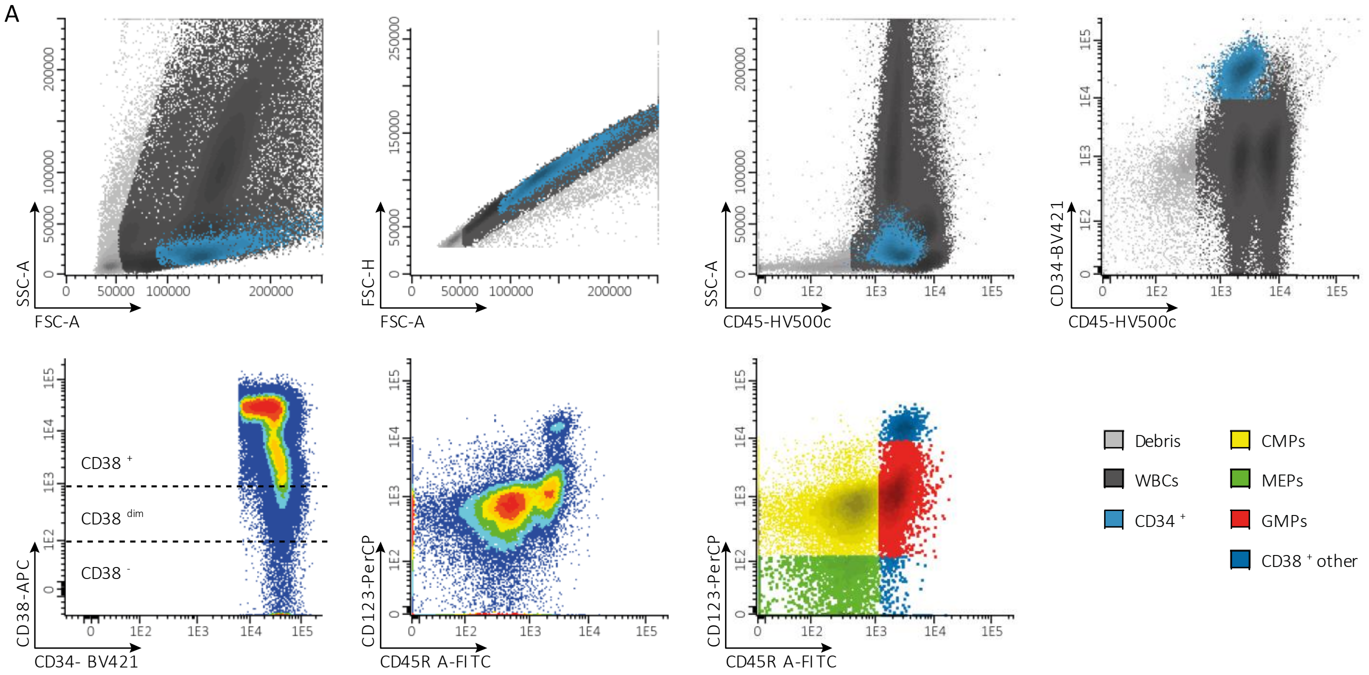

<u>Figure S2</u> Gating strategy

The FACS plots illustrate the gating strategy of the total CD34$^+$ compartment and distinct stem and progenitor cell subsets for NBM05. Flow cytometry data were manually analyzed using Infinicyt software (Cytognos). The remaining mature erythrocytes, debris and doublets were excluded in FSC-A/SSC-A and FSC-A/FSC-H plots. Within the white blood cells (WBCs), the CD34$^+$ compartment was identified based on intermediate CD45 and bright CD34 expression (in blue) and subsequently back-gated to homogeneity in FSC-A/SSC-A and CD45/SSC-A plots (as shown in blue in the first two plots). The CD34$^+$ compartment was divided into CD34$^+$CD38$^-$ cells, CD34$^+$CD38^{dim} cells and CD34$^+$CD38$^+$ progenitors using density plots and CD38 calibration particles (Spherotech, not shown). The CD34$^+$CD38$^+$ progenitors were classified into CD45RA$^-$CD123$^+$ common myeloid progenitors (CMPs), CD45RA$^+$CD123$^+$ granulocyte-monocyte progenitors (GMPs) and CD45RA$^-$CD123$^-$ megakaryocyte-erythroid progenitors (MEPs). The remaining CD34$^+$CD38$^+$ progenitors that were not classified as CMPs, GMPs and MEPs comprise, amongst others, common lymphoid cells and more mature subsets. Note that colors in the 5th and 6th panel indicate cell density, whereas the colors in the 7th panel indicate cell populations.

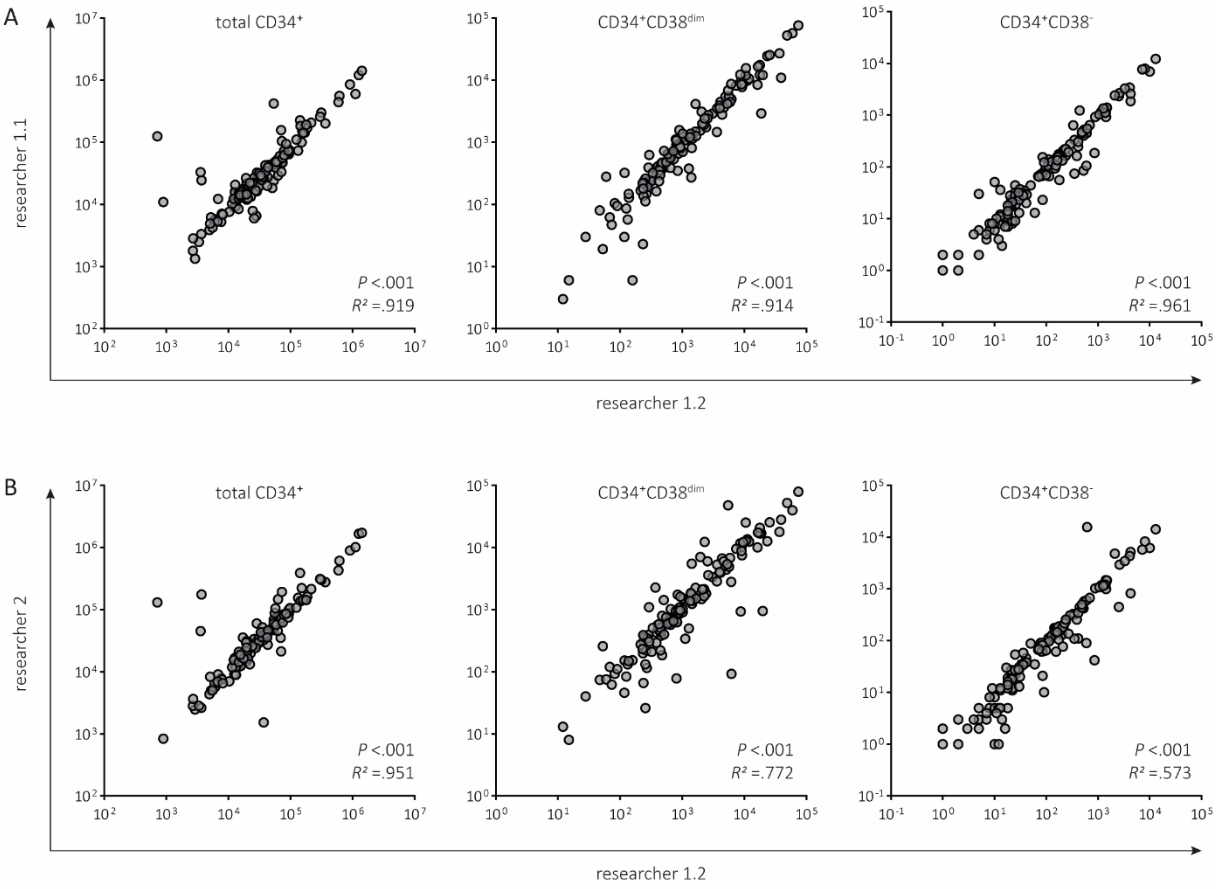

C

researcher 1.2 \ researcher 1.1	IA-HSC$^-$	IA-HSC$^+$	<10 CD38$^-$ cells
IA-HSC$^-$	98	0	4
IA-HSC$^+$	2	26	0
<10 CD38$^-$ cells	1	0	8

intra-observer variability = 5.04%

D

researcher 1.2 \ researcher 2	IA-HSC$^-$	IA-HSC$^+$	<10 CD38$^-$ cells
IA-HSC$^-$	93	0	9
IA-HSC$^+$	0	28	0
<10 CD38$^-$ cells	0	0	9

inter-observer variability = 6.47%

Figure S3 Validation of the gating strategy

The data were analyzed twice by researcher 1 (referred as 1.1 and 1.2) and once by an additional researcher 2 to assess intra- and inter-observer variability. Panel (**A**) shows strong correlations between the absolute CD34$^+$ numbers. (**B**) There is a significant relationship between the analyses of researcher 1 and 2, although less strong for CD34$^+$CD38^{dim} and CD34$^+$CD38$^-$ numbers due to their low abundance and the subjectivity of discriminating CD38^{dim} from CD38$^-$ and CD38$^+$.

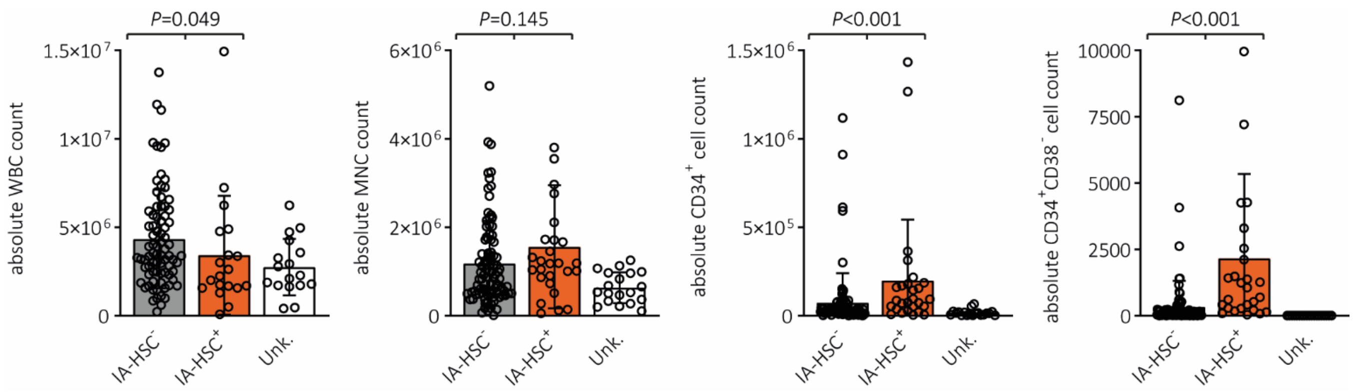

Figure S4 Measured CD34$^+$ cell counts

Boxplots illustrating the absolute number of WBCs and CD34$^+$ cell subsets in IA-HSC$^-$ and IA-HSC$^+$ BM samples from all patients and controls. The number of CD34$^+$ and CD34$^+$CD38$^-$ cell numbers was higher in IA-HSC$^+$ as compared to IA-HSC$^-$ BM samples, whereas the number of measured WBCs (only shown for freshly processed samples) was lower. To account for an underestimation of IA-HSCs numbers in small samples, a limit of detection (LoD) and limit of quantification (LoQ) was set at 10 and 30 CD34$^+$CD38$^-$ cells, respectively. The intra- and inter-observer variability of CD34$^+$ cell subset numbers are summarized in Figure S3.

Table S3A Univariate survival analysis

	LFS time	EFS time	OS time
IA-HSCs (yes vs. no)	***P*=0.004**<br>HR=25 (2.9-218)	***P*<0.001**<br>HR=49 (6.1-387)	***P*<0.001**<br>HR=9.3 (3.2-27)
IA-HSC (%)[1]	***P*=0.065**<br>HR=1.02 (1.00-1.04)	***P*=0.002**<br>HR=1.03 (1.01-1.05)	***P*<0.001**<br>HR=1.03 (1.02-1.05)
$CD34^+CD38^-$ (%)	***P*=0.055**<br>HR=2.2 (0.98-5.13)	*P*=0.153	*P*=0.957
$CD34^+CD38^{dim}$ (%)	***P*=0.029**<br>HR=1.4 (1.03-1.81)	***P*=0.075**<br>HR=1.3 (0.98-1.6)	*P*=0.883
CMPs (%)	*P*=0.859	*P*=0.522	*P*=0.992
GMPs (%)	*P*=0.455	***P*=0.006**<br>HR=1.09 (1.02-1.15)	*P*=0.842
MEPs (%[1])	*P*=0.620	*P* =0.685	*P*=0.125
BM blasts (%)<br>>2 - <5 vs. ≤2<br>≥5 - ≤10 vs. ≤2<br>>10 vs. ≤2	*P*=0.291	*P*=0.695	*P*=0.184
Hemoglobin (g/dL)<br>8 - <10 vs. ≥10<br><8 vs. ≥10	*P*=0.305	*P*=0.726	*P*=0.341
Platelets ($\cdot 10^9$/L)<br>50 - <100 vs. ≥100<br><50 vs. ≥100	***P*=0.095**<br>HR=11 (1.2-99)<br>HR=4.1 (0.25-65)	***P*=0.043**<br>HR=6.6 (1.3-35)<br>HR=7.7 (1.4-42)	***P*=0.064**<br>HR=2.4 (0.72-8.0)<br>HR=3.9 (1.2-13)
CCSS<br>L vs. VL<br>INT vs. VL<br>H vs. VL<br>VH vs. VL	*P*=0.109	***P*=0.010**<br>HR=$9.7 \cdot 10^2$ (0-$7.2 \cdot 10^{102}$)<br>HR=$5.6 \cdot 10^2$ (0-$4.1 \cdot 10^{102}$)<br>HR=1.2 (0-$4.4 \cdot 10^{123}$)<br>HR=$1.5 \cdot 10^4$ (0-$1.1 \cdot 10^{104}$)	***P*=0.001**<br>HR=$7.5 \cdot 10^2$ (0-$4.8 \cdot 10^{104}$)<br>HR=$7.9 \cdot 10^2$ (0-$5.0 \cdot 10^{104}$)<br>HR=0.99 (0-$4.9 \cdot 10^{124}$)<br>HR=$1.3 \cdot 10^4$ (0-$8.6 \cdot 10^{105}$)
IPSS-R<br>L vs. VL<br>INT vs. VL<br>H vs. VL<br>VH vs. VL	*P*=0.198	***P*=0.038**<br>HR=$1.2 \cdot 10^4$ (0-$8.3 \cdot 10^{105}$)<br>HR=$7.6 \cdot 10^4$ (0-$5.3 \cdot 10^{106}$)<br>HR=$3.8 \cdot 10^4$ (0-$2.6 \cdot 10^{106}$)<br>HR=$21 \cdot 10^4$ (0-$1.5 \cdot 10^{107}$)	***P*=0.012**<br>HR=$1.1 \cdot 10^4$ (0-$9.1 \cdot 10^{99}$)<br>HR=$6.2 \cdot 10^4$ (0-$4.9 \cdot 10^{100}$)<br>HR=$3.0 \cdot 10^4$ (0-$2.4 \cdot 10^{100}$)<br>HR=$2.1 \cdot 10^5$ (0-$1.6 \cdot 10^{101}$)
Therapy<br>LEN vs. SC<br>AZN vs. SC<br>CTx/SCT vs. SC	***P*=0.041**<br>HR=0<br>HR=3.9 (0.4-43)<br>HR=14 (2.3-89)	***P*=0.001**<br>HR=0.8 (0.1-8.4)<br>HR=8.7 (1.6-46)<br>HR=20 (4.2-95)	*P*=0.404

P values <0.100 are indicated by bold font and presented with the hazard ratio (95% CI). For categorical parameters, the first category was used as reference. The IA-HSC percentages are relative from total $CD34^+CD38^-$ cells, whereas $CD34^+CD38^-$ and $CD34^+CD38^{dim}$ cell percentages are relative from MNCs. [1] The prognostic value of IA-HSC percentages was estimated in samples meeting the LoQ threshold, i.e. ≥30 $CD34^+CD38^-$ cells (Figure S1). Abbreviations: VL, very low-risk; L, low-risk; INT, intermediate-risk; H, high-risk; VH, very high-risk; LEN, lenalidomide; AZA, azacitidine; CTx, chemotherapy; SC, supportive care; SCT, stem cell transplantation.

Table S3B Multivariate survival analysis

Model	Parameters	LFS time, *P*-value	EFS time, *P*-value	OS time, *P*-value
1A	IA-HSCs (yes vs. no)	**0.005**	**<0.001**	**<0.001**
	$CD34^+CD38^-$ (%)	0.972	0.489	0.462
1B	IA-HSCs (yes vs. no)	**0.006**	**<0.001**	**<0.001**
	$CD34^+CD38^{dim}$ (%)	0.954	0.397	0.322
1C	IA-HSCs (yes vs. no)	**0.002**	**<0.001**	**<0.001**
	GMPs (%)	0.316	0.147	0.865
1D	IA-HSCs (yes vs. no)	**0.007**	**0.001**	**0.001**
	Platelets ($\cdot 10^9$/L)	0.206	0.530	0.696
	50 - <100 vs. ≥100			
	<50 vs. ≥100			
1E	IA-HSCs (yes vs. no)	**0.010**	**0.001**	**0.030**
	CCSS	0.748	0.732	0.200
	L vs. VL			
	INT vs. VL			
	H vs. VL			
	VH vs. VL			
1F	IA-HSCs (yes vs. no)	**0.007**	**0.001**	**0.007**
	IPSS-R	0.468	0.770	0.424
	L vs. VL			
	INT vs. VL			
	H vs. VL			
	VH vs. VL			
1G	IA-HSCs (yes vs. no)	**0.034**	**0.003**	**<0.001**
	Therapy	0.243	**0.049**	0.856
	LEN vs. SC			
	AZN vs. SC			
	CTx/SCT vs. SC			

P values <0.100 are indicated by bold font. For categorical parameters, the first category was used as reference. The IA-HSC percentages are relative from total $CD34^+CD38^-$ cells, whereas $CD34^+CD38^-$ and $CD34^+CD38^{dim}$ cell percentages are relative from MNCs. Although the variable therapy added significant prognostic value to the presence of IA-HSCs, no further models are shown because of the low number of events. Abbreviations: VL, very low-risk; L, low-risk; INT, intermediate-risk; H, high-risk; VH, very high-risk; LEN, lenalidomide; AZA, azacitidine; CTx, chemotherapy; SC, supportive care; SCT, stem cell transplantation.

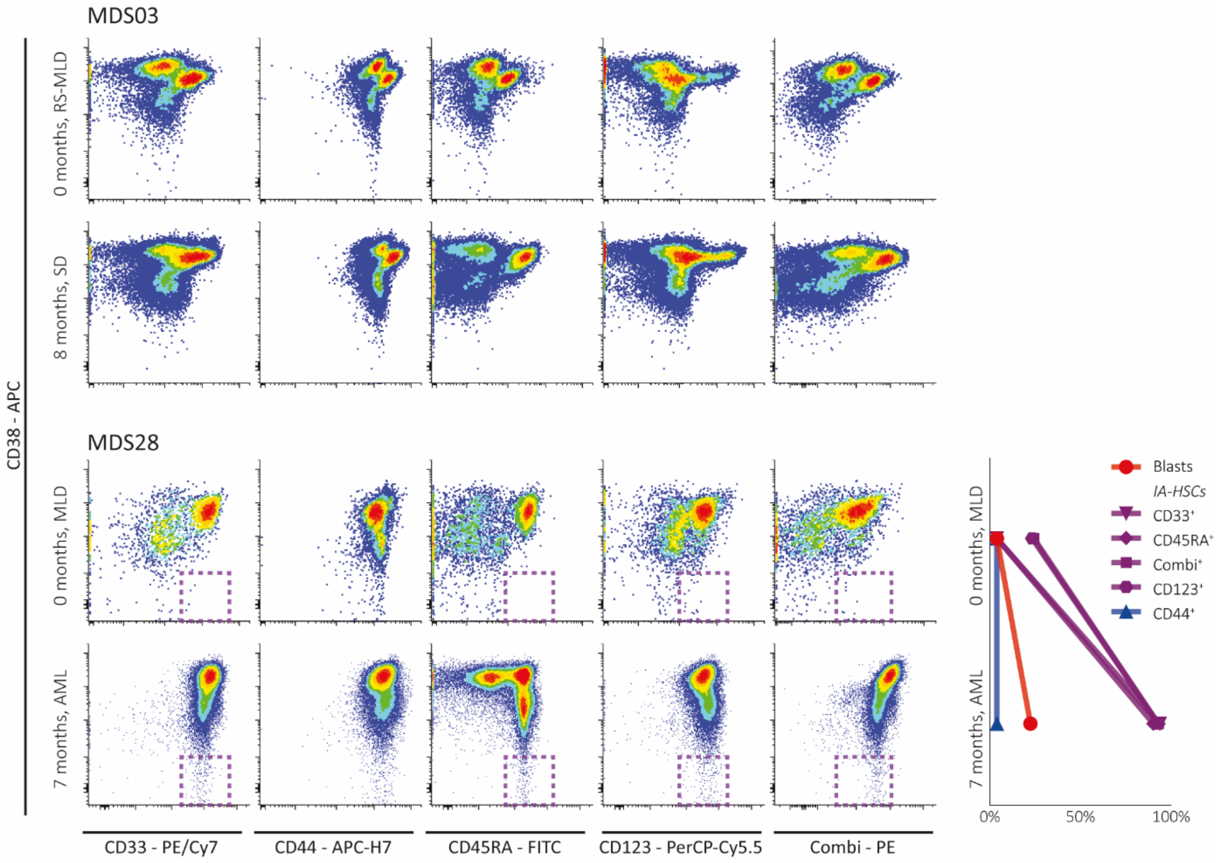

Figure S5 Analysis of stem cells in sequential bone marrow samples

Sequential BM samples were available in some MDS patients. This figure demonstrates an example of a MDS patient with a stable disease and disease progression. The expression of CD33, CD44, CD45RA, CD123 and Combi markers on the total $CD34^+$ HSPC compartment is presented. In MDS03, the second BM sample collected 8 months after diagnosis confirmed a stable disease (SD) with preservation of HSCs with immunophenotypes similar to the profile at the time of diagnosis. In MDS28, leukemic progression co-occurs with an expansion of IA-HSCs.

Table S4 Mutations in BM-MNCs from a subsampled MDS patient cohort

ID	IA-HSC%[1]	IPSS-R	Outcome	Gene	VAF	Function
MDS13	0.000[2]	L	OS 33 mo	SRSF2	48%	RNA splicing
MDS20	0.000	L	OS >46 mo	CALR	27%	chaperoning and calcium homeostasis
MDS43	0.000[3]	H	OS >17 mo	NRAS	48%	signal transduction
				ASXL1	46%	chromatin remodeling
				ETNK1	46%	phosphatidylethanolamine synthesis
				RUNX1	33%	transcription
MDS28	0.001	L	LFS 7 mo	-		
MDS73	0.045	L	EFS 3 mo	DNMT3A	51%	DNA methylation
				IDH2	47%	DNA methylation
				SRSF2	46%	RNA splicing
MDS44	0.007	INT	OS >1mo	SETPB1	44%	histone methylation
				SRSF2	47%	pre-mRNA splicing
MDS9	0.012	INT	EFS 18 mo	SRSF2	51%	RNA splicing
				IDH2	43%	DNA methylation
				ASXL1	42%	RNA splicing
MDS10	0.036	INT	EFS 6 mo	TP53	81%	tumor suppressor
MDS17	0.306	INT	OS 9 mo	SFRB1	49%	RNA splicing
				SRSF2	46%	RNA splicing
				ASXL1	35%	chromatin remodeling
				RUNX1	15%	transcription
				TET2	1%	DNA methylation
MDS8	0.026	VH	EFS 3 mo	TP53	35%	tumor suppressor

Patients are ranked by the percentage of IA-HSCs and IPSS-R. Using the TruSight panel (Table S2), genomic variants were detected in BM-MNCs from all MDS patients except for MDS28. [1] The percentage of IA-HSCs relative to MNCs. [2] In MDS13, the limit of detection of IA-HSCs was not reached as only 5 $CD34^+CD38^-$ cells were measured. [3] Although the number of mutations found in MDS43-derived MNCs may question the sensitivity of flow cytometric detection of IA-HSCs, it is important to note that 94 $CD34^+CD38^-$ cells, a relatively high number, were measured in this patient sample.

In vivo xenotransplantation

Cryopreserved BM-MNCs from 10 MDS patients (IA-HSC$^-$, n = 3; IA-HSC$^+$, n = 7) at the time of diagnosis were thawed as described above. Due to the scarcity of bone marrow tissue, the absence of IA-HSCs in one MDS sample (UPN: MDS75) was defined using a diagnostic MDS panel including CD7, CD11b, CD33, CD56 and CD123 but not CD44 and CD45RA. Thawed BM-MNCs were washed and suspended in PBS with 0.1% HSA. To deplete T cells, BM-MNCs were incubated with CD3 microbeads (Miltenyi) and loaded onto MACS columns following manufacturers' instructions. The CD3 depletion failed in four experiments (Figure S6). We decided to inject all xenografts irrespective of the CD3$^+$ percentage and to account for xenograft variances afterwards as described below. Female NSG (NOD/SCID gamma) mice were purchased from the Jackson Laboratory (Harbor, MN). One day before transplantation, the 9-weeks old mice were sublethally irradiated (200 cGy). The xenografts were injected via the tail vein in five mice per MDS patient. The division of xenografts in mice was not randomized nor blinded. Eight mice were sacrificed ahead of the experimental endpoint because of abnormal behavior directly after irradiation (n=1), severe weight loss (n=5) and intra-abdominal masses (n=2). The remaining 42 mice were sacrificed at 20 weeks post-transplantation. Peripheral blood (PB), the spleen and BM were harvested. All compartments were stained for the presence of murine CD45 (muCD45) and human CD45 (huCD45) in addition to lineage- and leukemia-associated markers (Table S1B). Mice with a minimum of 0.5% cells expressing huCD45 and another human cell marker apart from CD3 (CD19, CD33, CD34) relative to the total CD45 compartment (sum muCD45 and huCD45) were considered as engrafted. Mice showing human engraftment were studied for the presence of leukemic cells. Leukemic engraftment was suggested by an increased blast count in addition to the presence of leukemia-associated immunophenotypes corresponding to the original patient sample. Engraftment percentages were related to both MDS patient characteristics and xenograft variances, including the number of injected CD3$^+$ T cells and total BM-MNCs (Figure S6).

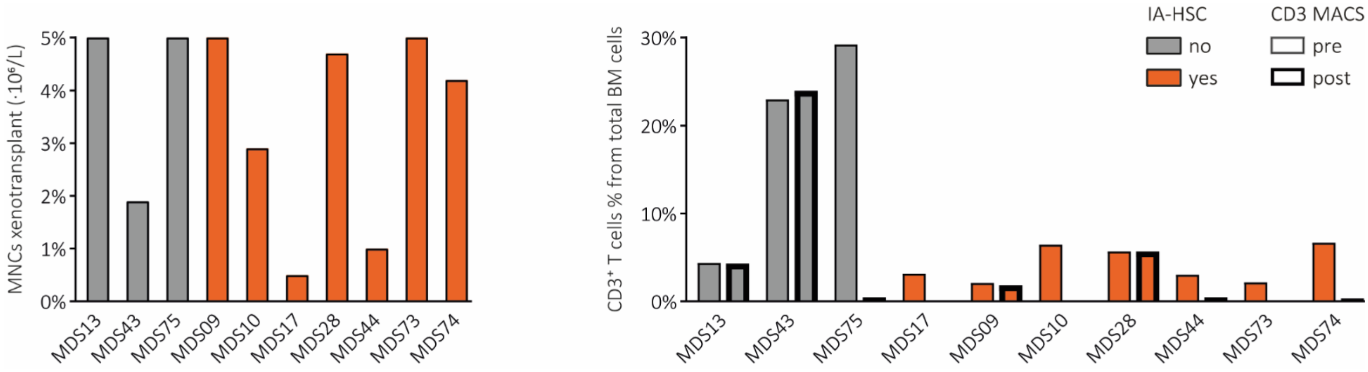

167 Figure S6 Xenograft counts

168 The number of frozen-thawed BM-MNCs per MDS xenograft and percentage of CD3⁺ T cells before (pre)

169 and after CD3 depletion (post) using MACS.

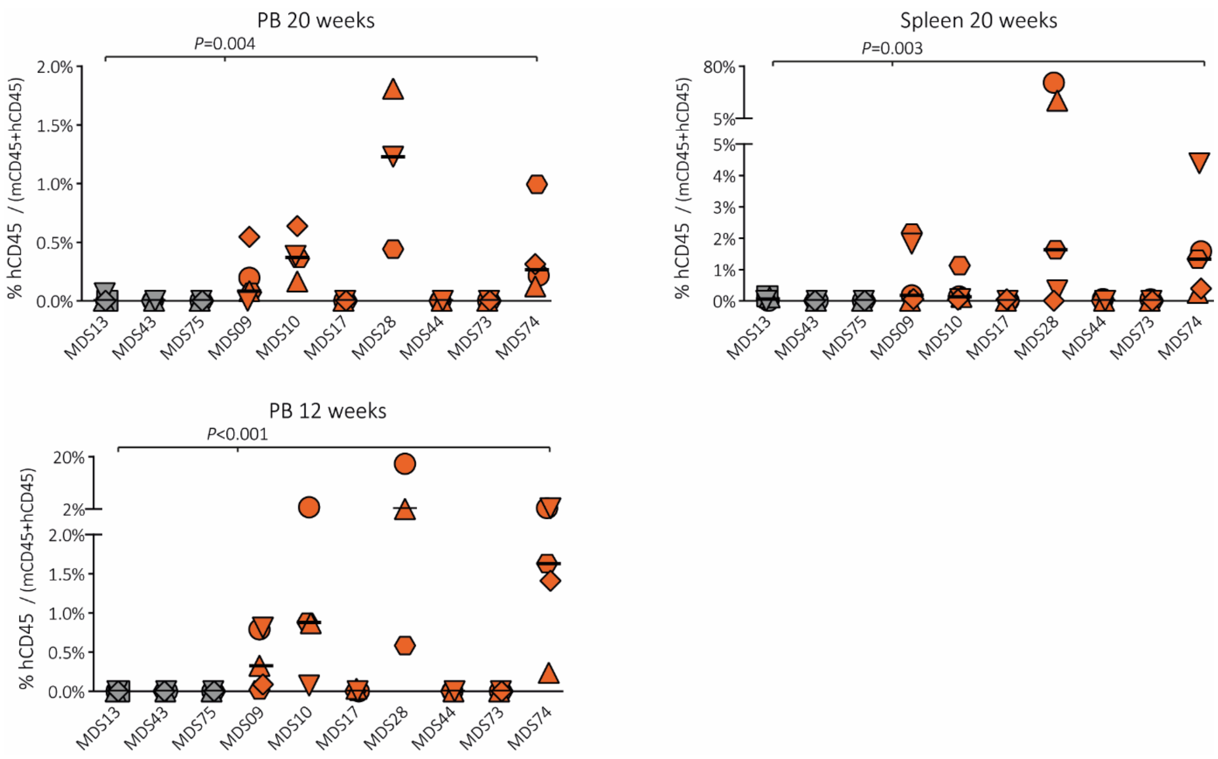

Figure S7 Human engraftment in spleen and peripheral blood

Mice injected with IA-HSC⁻ or IA-HSC⁺ MDS samples are indicated by the grey and orange color, respectively. The percentages of huCD45⁺ cells are plotted as median with 95%CI. At 12 weeks, PB was drawn via the tail vein to assess human engraftment. At 20 weeks, mice were sacrificed and the PB, BM and spleen were studied. The difference in huCD45⁺ cells between IA-HSC⁺ and IA-HSC⁻ transplanted mice was tested for statistical significance using the Mann-Whitney U test, as indicated by the *P*-values.

References

1. Zeijlemaker W, Kelder A, Oussoren-Brockhoff YJ, Scholten WJ, Snel AN, Veldhuizen D, et al. A simple one-tube assay for immunophenotypical quantification of leukemic stem cells in acute myeloid leukemia. Leukemia 2016; 30: 439-46.

2. Hanekamp D, Snel AN, Kelder A, Scholten WJ, Khan N, Metzner M, et al. Applicability and reproducibility of acute myeloid leukaemia stem cell assessment in a multi-centre setting. Br J Haematol 2020; 190: 891-900.

3. van der Pol MA, Pater JM, Feller N, Westra AH, van Stijn A, Ossenkoppele GJ, et al. Functional characterization of minimal residual disease for P-glycoprotein and multidrug resistance protein activity in acute myeloid leukemia. Leukemia 2001; 15: 1554-63.

4. van Rhenen A, Feller N, Kelder A, Westra AH, Rombouts E, Zweegman S, et al. High stem cell frequency in acute myeloid leukemia at diagnosis predicts high minimal residual disease and poor survival. Clin Cancer Res 2005; 11: 6520-7.
